# The Effect of Saturated Fatty Acids on Methanogenesis and Cell Viability of *Methanobrevibacter ruminantium*

**DOI:** 10.1155/2013/106916

**Published:** 2013-04-28

**Authors:** Xuan Zhou, Leo Meile, Michael Kreuzer, Johanna O. Zeitz

**Affiliations:** ^1^ETH Zurich, Institute of Agricultural Sciences, Universitaetstrasse 2, 8092 Zurich, Switzerland; ^2^ETH Zurich, Institute of Food, Nutrition and Health, Schmelzbergstrasse 7, 8092 Zurich, Switzerland

## Abstract

Saturated fatty acids (SFAs) are known to suppress ruminal methanogenesis, but the underlying mechanisms are not well known. In the present study, inhibition of methane formation, cell membrane permeability (potassium efflux), and survival rate (LIVE/DEAD staining) of pure ruminal *Methanobrevibacter ruminantium* (DSM 1093) cell suspensions were tested for a number of SFAs. Methane production rate was not influenced by low concentrations of lauric (C_12_; 1 **μ**g/mL), myristic (C_14_; 1 and 5 **μ**g/mL), or palmitic (C_16_; 3 and 5 **μ**g/mL) acids, while higher concentrations were inhibitory. C_12_ and C_14_ were most inhibitory. Stearic acid (C_18_), tested at 10–80 **μ**g/mL and ineffective at 37°C, decreased methane production rate by half or more at 50°C and ≥50 **μ**g/mL. Potassium efflux was triggered by SFAs (C_12_ = C_14_ > C_16_ > C_18_ = control), corroborating data on methane inhibition. Moreover, the exposure to C_12_ and C_14_ decreased cell viability to close to zero, while 40% of control cells remained alive after 24 h. Generally, tested SFAs inhibited methanogenesis, increased cell membrane permeability, and decreased survival of *M. ruminantium* in a dose- and time-dependent way. These results give new insights into how the methane suppressing effect of SFAs could be mediated in methanogens.

## 1. Introduction 

Methane (CH_4_) as a potent greenhouse gas is among the most important drivers of compositional changes of atmospheric gas and thus global warming [1]. Agricultural CH_4_ emissions account for about 50% of total CH_4_ from anthropogenic sources, where the single largest one is from enteric fermentation in ruminant livestock [2]. Methane is generated by a subgroup of the Archaea, the methanogens, which are, in the ruminant's fore-stomach (rumen), dominated by *Methanobrevibacter *[3]. At undisturbed rumen function, proteins and polymeric carbohydrates as main components of the diet are degraded by microorganisms and fermented mainly to volatile fatty acids (VFAs), ammonia, hydrogen (H_2_), and carbon dioxide (CO_2_). Ruminal methanogens primarily utilize H_2_ as energy source to reduce CO_2_ to CH_4_ in a series of reactions that are coupled to ATP synthesis [4, 5]. As CH_4_ cannot be utilized in the metabolism of the animal, ruminal methanogenesis also impairs feed conversion efficiency and represents a significant waste of energy (2% to 12% of energy intake; [6]). 

Therefore, inhibition of ruminal methanogenesis should be approached by various interventions. Among the most effective are dietary medium- and long-chain saturated fatty acids (SFAs). Nonesterified lauric acid (C_12_) was reported to have a particularly high potential in suppressing ruminal methanogenesis, followed by myristic acid (C_14_) [7–9]. By contrast, long-chain SFAs (LCFAs) such as palmitic acid (C_16_) and stearic acid (C_18_) were not effective in suppressing ruminal methanogenesis *in vitro* [7, 10]. The production of CH_4_ by pure, growing, cultures of *M. ruminantium*, a dominant ruminal methanogen [3], was found to be inhibited by the addition of unsaturated [11, 12] and saturated medium-chain (C_12_–C_16_; [12]) fatty acids. When testing the nonruminal methanogens *Methanothermobacter thermoautotrophicus* and *Methanococcus voltae*, C_12_ and C_14_ were found to inhibit methanogenesis as well [13]. However, systematic studies on dose-response relationships with SFAs on methanogenesis in pure ruminal methanogen cultures are missing. Besides, it is unclear why long-chain SFAs do not inhibit methanogenesis and if this is related to the low solubility of these long-chain SFAs at temperatures below 40°C [13, 14]. Furthermore, although fatty acids (FAs) are known to have antimicrobial and cytotoxic properties [15] and are used by a wide range of organisms like humans [16], molluscs [17], and brown algae [18] to defend against pathogens, the mechanisms which lead to the inhibition effect are still not definitely known. Several mechanisms have been proposed [15]. The primary target of the action seems to be the microbial cell membrane and various essential processes that occur within and at the membrane [15]. Fatty acids, including C_12_, C_14_, C_16_, and C_18_, have been shown to pass protein-free phospholipid bilayers in their unionized form [19]. Saturated and unsaturated fatty acids may be adsorbed by bacterial cell membranes [20], damage the bacterial cell membrane as determined by loss of potassium (K^+^) [21], ATP, and proteins [16] and by electron microscopy [22, 23], and play a role in cell death [22, 24, 25]. As the composition of the cell envelope of methanogens is fundamentally different from the bacterial cell envelope, and the methanogens are phylogenetically and physiologically distinct from all other cell types [26], the mechanisms of FA action on methanogens may differ from that valid for other organisms. However, since the methanogen cell envelope normally acts as a diffusion barrier between the cytoplasm and the extracellular medium, it might also represent a key point for the identification of inhibitor targets. Therefore, we hypothesized that membrane integrity is disturbed and leakage of cell metabolites including inorganic ions such as K^+^ occurs through the interaction of the SFA with the cell membrane lipids and that this results in an impaired cell survival. Like in most prokaryotes, K^+^ is accumulated in the cytoplasm of methanogens in exchange for Na^+^ [27]. 

In the present study, pure cultures of *M. ruminantium* were treated with pure nonesterified SFAs in order to exclude all confounding factors such as interactions between feed, minerals, and microbes occurring *in vivo* or with rumen fluid *in vitro*. The aims of the present study were (i) to investigate the relationship between SFA type and dosage and the inhibition of methanogenesis in nongrowing cells, that is, cell suspensions, and (ii) to get first insights into the modes of action underlying in this process. In detail, K^+^ efflux was used as an indicator of membrane integrity. Finally, cell survival was monitored using the LIVE/DEAD BacLight Kit which has been successfully used in Archaea before [28, 29].

## 2. Materials and Methods

### 2.1. Strain and Growth Conditions

A pure culture of *M. ruminantium* M1 (DSM 1093) was obtained from the “Deutsche Sammlung von Mikroorganismen und Zellkulturen,” Braunschweig, Germany. It was anaerobically cultivated in the strain-specific cultivation medium 119 prepared according to DSMZ (http://www.dsmz.de) in 120 mL serum bottles, which were sealed with butyl rubber stoppers (20 mm size; 2048-11800, Bellco, Vineland, USA) and aluminum seals (2048-11020, Bellco). Reagents for the media were dissolved in boiled oxygen-deprived distilled water and stirred on a magnetic stirrer overnight in an anaerobic chamber (Coy Laboratory Products, Grasslake, USA). Heat stable solutions of media ingredients were sterilized in a batch autoclave (Sauter, Belimed Sauter AG, Sulgen, Switzerland) for 20 min at 121°C. Heat susceptible solutions, that is, vitamins, sodium formate, and SFA, were filtrated through a 0.2 *μ*m Minisart-plus filter (Sartorius AG, Göttingen, Germany). Ruminal fluid was obtained from a rumen-cannulated cow, filtered through four layers of medicinal gauze (REF 200137, Novamed, Jerusalem, Israel) and then centrifuged twice for 15 min at 4,000 ×g (Varifuge K, Heraeus, Osterode, Germany). The supernatant was adjusted to pH 7.0 with HCl and NaOH, gassed with N_2_ to an atmospheric pressure of 150 kPa, autoclaved, and stored at −20°C for up to 6 months, before being used to prepare the media. Aliquots of the prepared medium were filled into 250-mL bottles, closed with rubber septa, gassed with N_2_ to atmospheric pressure of 250 kPa, autoclaved, and stored either at 4°C for 8 weeks or at −20°C for up to 6 months before being used. *M. ruminantium* was grown under atmospheric pressure of 250 kPa of a CO_2_/H_2_ mixture (20 : 80) (Pangas AG, Dagmarsellen, Switzerland). The gas mixture in the headspace was renewed every 24 h and 3 mL precultures were transferred to 27 mL fresh medium every four days. The culture bottles were incubated in horizontal position in an incubation shaker (Incu Shaker 10 L, Benchmark, Korea) at 37°C with a shaking speed of 150 rpm. Growth of the cultures was monitored by recording CH_4_ production, gas consumption, and optical density. A volume of 0.15 mL of gas was collected from the headspace of the cultivation bottle with a gas-tight syringe (Hamilton, model 1725/RN 250 mL, Fisher Scientific AG, Wohlen, Switzerland), and its CH_4_ concentration was analyzed with a gas chromatograph (model 6890N, Agilent Technologies, Santa Clara, CA, USA) equipped with a flame ionization detector operated at 250°C and a 234 mm × 23 mm column (80/100; 166 mesh; Porapak Q, Fluka Chemie AG, Buchs, Switzerland). Overpressure in the cultivation bottles was detected with a manometer (GDH 200-13, Greisinger Electronic GmbH, Regenstauf, Germany). One milliliter of culture liquid was collected in acrylic absorption cuvettes (1 cm path length; (VWR, Leuven, Germany)), and its optical density was measured at 600 nm (OD_600_) with a UV-160A recording spectrophotometer (Shimadzu, Kyoto, Japan). The growth phases distinguished were lag, exponential, stationary, and death phase. Prior to each experiment, methanogens were inoculated into fresh medium with 3 mL of pre-culture in their early to mid-exponential growth phase. 

### 2.2. Experiment 1

 Lauric acid, C_14_, C_16_, and C_18_ (≥97% purity) were obtained from Sigma-Aldrich, Buchs, Switzerland, to be used as experimental supplements. Stock solutions were prepared by dissolving the SFA in the sterile-filtered solvent dimethyl sulfoxide (DMSO) (Sigma-Aldrich) to reach concentrations of 1, 3, 5, 10, and 30 mg/mL (C_12_ to C_16_) as well as 50 and 80 mg/mL (C_18_). They were stored at room temperature before supplementation. The C_18_ solution to be applied later at 50°C was heated to 50°C before use.

As OD_600_ was used to estimate cell dry matter (DM) concentration in growing cultures prior to harvesting, a regression line between OD_600_ and cell DM concentration was established before the start of the experiment. Seventeen bottles of medium were prepared and inoculated with *M. ruminantium* as described before. From three bottles each, 21 mL of culture liquid were collected after 24, 48, 53, 72, 77, and 96 h covering the development from the early exponential growth phase to the stationary phase. Thereof, 1 mL was used for measurement of OD_600_, and 20 mL was dried at 70°C to constant weight in a 50 mL Falcon tube after the wet weight had been recorded in order to calculate culture DM content. The regression curve established from in total 17 OD/DM pairs (OD range: 0.348 to 0.986) was linear and reads DM (mg/mL) = 7.6092 × OD_600_ + 0.4754 (*R* ^2^ = 0.95). This relationship was used to adjust and equalize cell DM concentration in cell suspensions.

In order to prepare the experimental cell suspensions in an anaerobic chamber, always 20 mL of culture were harvested in the mid-exponential growth phase and transferred to two 50 mL sterilized Falcon tubes and centrifuged for 10 min at 3,000 ×g. The supernatant was discarded and the pellet was washed twice with an autoclaved phosphate buffer of pH 6.8 containing 0.025 M KH_2_PO_4_, 0.025 M K_2_HPO_4_, 0.5 mM titanium citrate, 0.1 M NaCl, and 1 mM MgCl_2_ [30]. Titanium citrate was prepared according to Jones and Pickard [31], by anaerobically adding 5 mL of a 15% titanium(III) chloride solution (Merck Millipore, Darmstadt, Deutschland) to 50 mL of 0.2 M sodium citrate solution, adjusting with a saturated sodium carbonate solution to pH 7, gassing the bottle with N_2_, followed by autoclaving. Syringes were used for all withdrawals. After washing, the cell pellet was then resuspended in the same buffer to a final concentration of 6 mg cell DM/mL adjusted with the help of the regression line relating OD and culture DM concentration. Under anaerobic condition, 1 *μ*L of the differently concentrated SFA stock solutions was added to 999 *μ*L cell suspensions in 25 mL serum bottles to reach concentrations of 1, 3, 5, 10, and 30 *μ*g/mL of C_12_, C_14_, and C_16_ as well as 50 and 80 *μ*g/mL of C_18_. The bottles were sealed with rubber stoppers (size 18D, 203018; Glasgerätebau Ochs, Lenglern, Germany), gassed to atmospheric pressure of 250 kPa with a CO_2_/H_2_ mixture (20 : 80) and stored on ice waiting for incubation start by putting into a waterbath (Julabo shake Temp, Merck, Switzerland) at set intervals due to time needed for GC measurement (3.2 min/sample). Cell suspensions were incubated at 37°C and 50°C (only C_18_) shaking suspensions at 150 rpm. Finally, suspensions where no SFA had been added were supplemented with either 1 *μ*L/mL DMSO, equal to the DMSO concentration in treatment groups (control group) or with 1 *μ*L/mL of the buffer (blank group). The CH_4_ concentration (mol %) was determined by gas chromatography after 1, 2, 5, and 24 h had passed. The CH_4_ production rate (*μ*mol CH_4_/mg cell DM per min) was calculated from bottle head space gas volume and the volume of CH_4_ produced. The amount of gas present in the bottles at the start of the experiment were set to 0.0023 mol as calculated from using the ideal gas law (*n* = *p* × *V*/*R* × *T*, where *p* is the sum of the overpressure of the gas in the bottle (150000 Pa) and the standard air pressure (96600 Pa for Zurich), *V* is the volume of the gas = 24 × 10^−6^ m^3^, *n* is the amount of gas in the bottle in mol, *T* is the temperature of the gas = 309.15 K, and *R* is the ideal gas constant = 8.314 J K^−1 ^mol^−1^). The amounts of CH_4_ produced in each bottle (*Y*; in mol) were calculated considering the stoichiometry of methanogenesis from H_2_ and CO_2_, that is, that 5 mol of gas are consumed to produce 1 mol of CH_4_ meaning *Y*/(0.0023 − 4 × *Y*) = mol% CH_4_  (*X*/100) and therefore *Y* = 0.0023*X*/(100 + 4*X*). 

For each SFA, a minimum of two independent cell suspension incubations were performed with freshly grown *M. ruminantium* culture, each performed at least in triplicate.

### 2.3. Experiments 2 and 3

 Cells were harvested as described before and resuspended to a final concentration of 6 mg cell DM/mL in K^+^-free buffer containing 0.025 M (NH_4_)_2_HPO_4_, 0.025 M NH_4_H_2_PO_4_, 0.01 M NaCl, 1 mM MgCl_2_, and 0.5 mM titanium citrate. Two resting cell suspension experiments were performed at 37°C as described before, and in Experiment 2, C_12_ was supplemented to final concentrations of 10, 15 and 30 *μ*g/mL, and in Experiment 3, C_12_, C_14_, C_16_ and C_18_ were added to reach a final concentration of 10 *μ*g/mL. After 3 h and 24 h of incubation, 300 *μ*L of cell suspension were transferred to a 2 mL centrifuge tube inside the anaerobic chamber, centrifuged at 10,000 ×g for 10 min, and the K^+^ concentration in the supernatant was analyzed by Inductively Coupled Plasma-Optical Emission Spectrometer (715-ES Radial ICP OES, Varian, Canada). A stock solution containing 1 mg/L KNO_3_ (Merck, Darmstadt, Germany) and 1% HNO_3_ in distilled water was used to prepare a calibration curve with concentrations of 0, 25, 50, 75, and 100 *μ*L/L. Samples were diluted 50-fold by using a diluter (Microlab 1000, Hanmilton, Martinsried, Germany) in 5 mL of total volume. The survival rate of *M. ruminantium* in cell suspensions after 3 and 24 h was assessed by using the LIVE/DEAD BacLight Bacterial Viability Kit for microscopy and quantitative assays (Kit L7012; Invitrogen GmbH, Darmstadt, Germany). The kit applied contained two fluorescent dyes: propidium iodide with red fluorescence penetrates cells with damaged membranes; SYTO 9 with green fluorescence accumulates only in living cells. Thus, undestroyed archaeal cells with intact membranes have green fluorescence, while cells with damaged membranes display red fluorescence. Occasionally, an intermediate ambiguous yellowish color has been observed which has been observed also in studies of others [28]. Cells showing this color have been categorized as living cells with damaged membrane but were not included into the category of living cells in the tables. Staining was performed according to the manufacturer's protocol with several modifications. An amount of 0.5 *μ*L of a 1 : 1 mixture of SYTO 9 and propidium iodide dyes was added to 100 *μ*L of cell suspension under aerobic conditions, mixed thoroughly and incubated at room temperature in the dark for 10 min. No washing was required before staining because background fluorescence was low in this experimental system, and oxygen exposure was minimized by this way. An amount of 5 *μ*L of the stained cell suspension was trapped between a microscope slide and an 18 mm square cover glass. All samples were examined at 600 and 1000 times magnification using a fluorescence microscope (BX60; Olympus GmbH, Voketswil, Switzerland) and a digital camera (FView; adapter U-CMAD, Olympus, Switzerland). Three locations on each sample were chosen and captured at random. Fluorescent micrographs (exposure time: 50 ms) of the very same sample section were taken applying appropriate filter sets for propidium iodide (wavelengths: excitation 530–545 nm, emission >610 nm) and SYTO 9 (excitation 440–470 nm, emission 525–550 nm) and using the digital image analysis software Analysis (Soft Image System GmbH, Münster, Germany). The two false-colored images of one sample section were combined using the same software, and dead and live cells were counted with Adobe Photoshop CS5 (Adobe, San Jose, USA). Postacquisition processing involved adjustments of the brightness/contrast to optimize the visualization of live and dead cells within the images. Viability was calculated as viability = *N*/*N*_0_ × 100, where *N*_0_ are the total fluorescence counts and *N* are the green fluorescence counts after 3 h and 24 h of reaction. Experiments 2 and 3 were performed in triplicate with three samples per treatment group and additionally, three samples for LIVE/DEAD staining and K^+^ leakage determination after 3 h.

### 2.4. Statistical Analysis

 For Experiment 1, analysis of variance was performed using the MIXED procedure of SAS (version 9.1 of 2003; SAS Institute Inc., Cary, NC) with treatment group and time point and its interaction as fixed factors and the repeated statement to compare control and SFA-supplemented cultures at each time-point. For Experiments 2 and 3, treatment group was considered as fixed and replicate as random factor to compare CH_4_ inhibition rate, K^+^ leakage and cell viability both at 3 and 24 h. The Bonferroni correction was used for multiple comparisons among means. Differences were declared statistically significant at *P* < 0.05. The results are presented as means ± standard errors. 

## 3. Results

### 3.1. Inhibition of Methane Production of *Methanobrevibacter ruminantium* by Saturated Fatty Acids as Depending on Dose in Experiment 1

**Figure 1:**
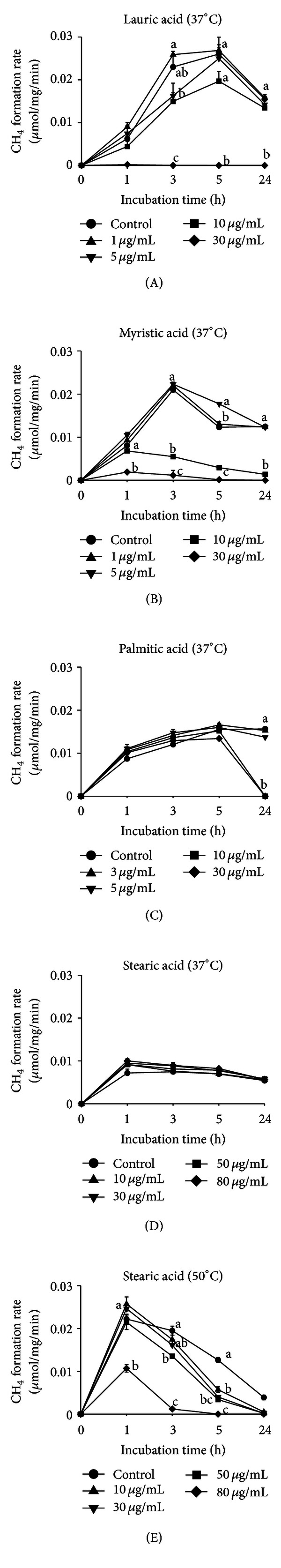
Methane production rate (*μ*mol/mg cell DM/min) in cell suspensions of *M. ruminantium* in K^+^-containing buffer (*n* = 3) in response to supplementation of different concentrations of lauric acid (A), myristic acid (B), palmitic acid (C), and stearic acid (D) at 37°C and of stearic acid at 50°C (E) (Experiment  1). Means within time point with unequal letters (a, b) are different at *P* < 0.05. Bars represent standard errors.

All SFAs investigated influenced CH_4_ production by *M. ruminantium* in a dose-dependent way, but the extent of the effect differed (Figure 1). In Figure 1, only one of the two incubations performed per SFA is shown (the other is given as Supplementary Figure  1), but values were similar between incubations. For C_12_, the CH_4_ production rate was inhibited in a dose-dependent way with (*μ*g/mL) 30 > 10 = 5 ≥ control ≥ 1 (incubation 1; Figure 1(a)) and 30 > 10 = 5 > 1 = control (incubation 2; Supplementary Figure  1). For C_14_, the sequence was 30 > 10 > 1 = control > 5 (incubation 1; Figure 1(b)) and 30 = 10 > 1 ≥ 5≥ control (incubation 2). The inhibitory pattern of C_16_ was different from C_12_ and C_14_; C_16_ needed more time to exert its influence: dosages of 10 and 30 *μ*g/mL inhibited the CH_4_ production rate at 24 h completely (incubation 1; Figure 1(c)) or by half (incubation 2) but not at earlier time points. Lower concentrations did not inhibit CH_4_ production during the measurement period. C_18_ was not effective at 37°C (Figure 1(d)) but at 50°C, a temperature closer to the melting point of C_18_ of 69°C. At 50°C, C_18_ decreased the CH_4_ production rate in a dose-dependent way after 5 h by 55% and 68% at 10 and 30 *μ*g/mL, respectively (incubation 1; Figure 1(e)). At 50 *μ*g/mL, the CH_4_ production rates started to decline even earlier and were decreased by 63% and 99% at 5 h and 24 h, and at 80 *μ*g/mL, by 52%, 94%, and 100% at 1 h, 3 h, and 5 h, respectively. 

### 3.2. Influence of Lauric Acid on Methane Production, K^+^ Leakage and Cell Viability in Experiment 2

 In K^+^-free buffer, the CH_4_ inhibitory pattern of C_12_ (Table 1) was similar as compared to Experiment 1 in K^+^-containing buffer; concentrations of ≥10 *μ*g/mL decreased the CH_4_ production rate very fast and, with 30 *μ*g/mL, stopped it completely after already 3 h. A quick increase in extracellular K^+^ concentration occurred in C_12_-treated groups after 3 h of incubation (Table 1). Especially in groups where 15 and 30 *μ*g/mL was added, extracellular K^+^ concentration reached its peak already at 3 h and did not increase as reaction time progressed. The viability of the *M. ruminantium* cells as verified using LIVE/DEAD staining at 3 h and 24 h after supplementation of 10, 15, and 30 *μ*g C_12_/mL is shown in Table 1. Although methanogenesis was completely inhibited and marked K^+^ leakage occurred in groups supplemented with 15 and 30 *μ*g C_12_/mL at 3 h, cell viability was still 27% and 29%, respectively, instead of being zero. Within 24 h, C_12_ caused more cell death.

### 3.3. Influence of Saturated Fatty Acids on Methane Production, K^+^ Leakage, and Cell Viability in Experiment 3

**Figure 2:**
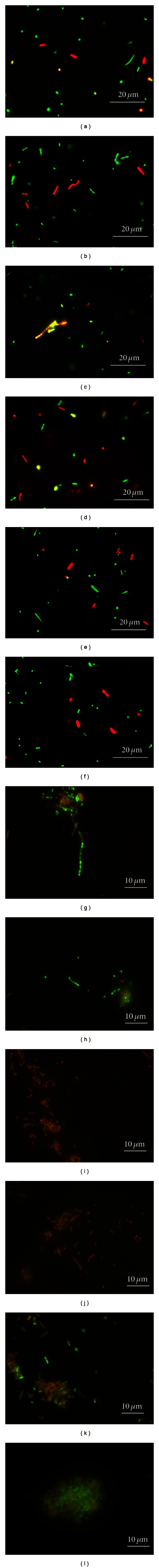
Fluorescence images illustrating cell viability of *M. ruminantium* cell suspensions exposed to different saturated fatty acids provided in a concentration of 10 *μ*g/mL in K^+^-free buffer and stained with the LIVE/DEAD BacLight Kit (Experiment  3). Green and red cells represent living and dead cells, respectively. Yellow cells were not categorized as living but included in total cell counts. (a–f) Images taken 3 h after SFA supplementation; (g–l) Images taken after 24 h. The images selected are representative for blank (a, g), control (b, h), C_12_ (c, i), C_14_ (d, j), C_16_ (e, k), and C_18_ (f, l).

All SFAs were supplemented in the same concentration (10 *μ*g/mL) in a single incubation to allow a direct comparison between SFAs (Table 2). C_12_ and C_14_ had a similar inhibitory effect on methanogenesis. Both immediately started displaying their influence. C_16_ needed more time and its effect was weaker than that of the former two SFAs, while C_18_ showed no effect at 37°C, which was consistent with the results of Experiment 1. The patterns of methanogenesis inhibition and K^+^ efflux were similar (Table 2). C_12_ and C_14_ also had the strongest effect of all SFAs tested in triggering K^+^ leakage, while C_16_ caused lower K^+^ efflux compared to C_12_ and C_14_, but the extracellular K^+^ concentration was higher (*P* < 0.05) than in control (Table 2). In summary, the K^+^ efflux was (in decreasing order): C_12_ = C_14_ > C_16 _> C_18_ > control. Interestingly, C_18_ showed no inhibitory effect on CH_4_ production rate but did cause K^+^ efflux (+23% as compared to the control after 3 h). C_12_ and C_14_ had the strongest effect on cell viability, as 57% and 64% of the cells were categorized as dead after 3 h, while in the C_16_ group only 32% of cells were dead or, as part of the cells were not red but yellow, damaged (Figure 2). At 24 h, nearly all cells treated with C_12_ and C_14_ were dead, compared to 60% of dead cells found in the control (Table 2 and Figure 2). Also in the C_16_ treatment 88% of cells were dead after 24 h, which implies that the inhibition of methanogenesis and K^+^ efflux are somehow correlated. C_18_ did not cause significant extra cell death when compared to the control group. 

## 4. Discussion

The antifungal and bactericidal properties of FA have been extensively investigated, and, as a generalization, the cell membrane seems to be the prime target to explain the effects of SFAs on the activity of cells and microorganisms [15]. However, studies on the effects of SFAs on pure cultures of ruminal methanogens are limited [12]. Although, finally, potential inhibitors of ruminal methanogenesis have to be evaluated with the mixed microbial community and in the presence of feeds, elucidating the SFA effects on individual methanogen species in the absence of further influencing factors is very important to differentiate direct and indirect SFA effects on methanogens and to identify the mechanisms which lead to the inhibition of methanogenesis by SFAs. 

### 4.1. Efficiency of Saturated Fatty Acids to Inhibit Methanogenesis in *Methanobrevibacter ruminantium*

In the present study, at first the effect of SFAs on CH_4_ production by cell suspensions of a major ruminal methanogen, *M. ruminantium, *was examined. The inhibition of methanogenesis in washed cell suspensions of *M. ruminantium* was getting more pronounced with decreasing chain length (C_12_ = C_14_ > C_16_ > C_18_) and increasing SFA concentration (1 to 80 *μ*g/mL suspension) or SFA/cell DM ratio (0.2 to 13 *μ*g/mg cell DM). Although cell inoculum each time was always applied by transferring the same volume using the microbes at almost the same growth phase and the cell suspensions were prepared by following the same protocol in each incubation, it seems that cell susceptibility varied between incubations, which also caused variability in the CH_4_ production patterns of the control groups (Figure 1 and Supplementary Figure 1). In agreement with studies performed at 35–38°C and neutral pH in cultures of ruminal and nonruminal methanogens and bacteria [12, 13, 32] and in sheep *in vivo* [33], the present data also indicate that C_12_ and C_14_ are the most effective SFAs. C_12_ had also been the most inhibitory representative of the SFAs against 12 Gram-positive microorganisms [32]. In the present study, the hydrophobic SFAs were dissolved in DMSO to guarantee distribution of SFA in the hydrophilic *M. ruminantium* cell suspension. Nevertheless, despite using DMSO, the SFA solubility was visually observed to decrease as SFA chain length increased. Solubility was especially weak when using C_18_ at 37°C where also no CH_4_ inhibition occurred. C_18_ was only inhibitory at 50°C, which corresponds with its increased solubility at this temperature. This supports the hypothesis that SFAs need to be at least partly dissolved in the buffer or medium to be able to exert an effect [13]. Further experiments have to investigate if the SFAs state (protonated versus dissociated) plays a role in *M. ruminantium*. Lowering the pH of the incubation medium has been shown to increase adsorption of SFAs onto bacteria and also their sensitivity against SFAs [20, 34]. The SFA concentrations needed to achieve a 50% reduction in CH_4_ formation rates were much lower in the present study than those required in the study of Henderson [12], where 0.5 g/L of C_12_ and C_14_ were necessary to reduce the growth rate of *M. ruminantium* by 50% compared to the control. This might have resulted either from the difference in metabolic state between cell cultures and cell suspensions or from differences in growth states before SFA supplementation and harvesting or both. Still, the SFA concentrations where a significant inhibition of methanogenesis occurred in the present study (10 to 80 *μ*g/mL) were in the same order of magnitude than those reported earlier (30 to 1000 *μ*g/mL) in growing methanogen cultures [12, 13, 20, 32]. This indicates that in cell cultures and cell suspensions generally the same type of effect occurs. Presumably, no cell growth occurred in the washed cell suspensions used due to the absence of nutrients needed for growth of *M. ruminantium*, like acetate and coenzyme M [35], and, in case of K-containing buffer, also nitrogen. Therefore, only CH_4_ production, that is, energy metabolism, was performed which indicates that the SFAs directly affect the process of CH_4_ formation. Each dose-response test had been repeated at least once and in both incubations in three replicates each to allow robust conclusions. Although the extent of the inhibition of methanogenesis by the different SFA concentrations was not exactly the same in the two incubations, the ranking and inhibition extent of the treatments with regard to the level of effect were coinciding. Slight variations in CH_4_ formation rates and peak times as well as in the SFA effects might be due to slight differences in growth phase between incubations when the cells being in their mid-exponential phase were harvested. 

### 4.2. Indications for Modes of Action of Saturated Fatty Acids

In the present study, the findings on K^+^ leakage, an indicator of a damaged membrane [21], indicate that the cell membrane permeability increases after SFA exposure. The integrity of the archaeal membrane is fundamental to maintain the chemiosmotic balance, which is essential for the membrane-associated energetic metabolism of cells [5, 26]. The K^+^ leakage also occurred concomitantly to the inhibition of methanogenesis which seems to have been followed by increasing occurrence of cell death. The K^+^ efflux in *M. ruminantium* responded to different SFAs and to different C_12_ concentrations similarly as the CH_4_ production rate. Accordingly, C_12_ and C_14_ triggered the largest K^+^ efflux and had the strongest inhibitory effects of all SFAs tested, and increasing C_12_ concentrations increasingly inhibited methanogenesis and promoted K^+^ efflux compared to the lower dosages. The LIVE/DEAD BacLight bacterial viability kit has been already shown to be a useful tool to indicate cell viability in Archaea [28, 29]. Although the CH_4_ production rate declined to zero, corroborated by heavy K^+^ leakage, in treatment groups supplemented with C_12_ and C_14_ at 3 h, and the percentage of cells with damaged membrane was significantly different to all other groups, it was not zero. It seems that cell death does not occur immediately but is delayed in time because after 24 h, and the cells in these two groups were nearly all dead. 

### 4.3. Conclusion

The inhibitory effect of SFAs on the production of the important greenhouse gas methane by *M. ruminantium *was demonstrated to be dependent on SFA concentration, SFA type, and incubation temperature (37°C versus 50°C). The present study showed for the first time with a ruminal methanogen, *M. ruminantium*, that supplementation of SFAs can also damage the cell membrane and trigger K^+^ efflux. The identification of the detailed mechanism on how SFAs are detrimental to the methanogens needs further studies. 

## Supplementary Material

Methane production rate (**µ**mol/mg cell DM/min) in cell suspensions of *M. ruminantium* in K^+^-containing buffer (n = 3) in response to supplementation of different concentrations of lauric acid (A), myristic acid (B), palmitic acid (C) and stearic acid (D) at 37°C and of stearic acid at 50°C (E) in the second incubation series. Means within time point with unequal letters (a, b) are different at *P* < 0.05. Bars represent standard errors.

## Figures and Tables

**Table 1:** Methane inhibition rate, K^+^ efflux, and cell viability in cell suspensions treated with C_12_ in different concentrations in Experiment 2 (*n* = 3; means ± standard error).

Time	3 h			24 h		
Treatment	K^+^(mg/L)	CH_4_ inhibition (%)^1^	Cell viability (%)^2^	K^+^(mg/L)	CH_4_ inhibition (%)^1^	Cell viability (%)^2^
Blank	12.8 ± 1.7^b^	20.7 ± 8.1^b^	75 ± 3^a^	16.0 ± 0.2^b^	13.5 ± 16.3^b^	56 ± 2^ab^
Control	11.1 ± 0.2^b^	—^b^	79 ± 2^a^	16.3 ± 0.4^b^	—^b^	61 ± 5^a^
10 *μ*g/mL	12.8 ± 0.3^b^	89.6 ± 5.1^a^	24 ± 5^b^	18.5 ± 0.1^a^	95.1 ± 2.5^a^	53 ± 7^ab^
15 *μ*g/mL	18.4 ± 0.2^a^	99.8 ± 0.1^a^	27 ± 4^b^	19.3 ± 0.2^a^	99.8± 0.1^a^	35 ± 4^bc^
30 *μ*g/mL	19.0 ± 0.3^a^	100.0 ± 0.1^a^	29 ± 6^b^	19.1 ± 0.4^a^	100 ± 0.0^a^	13 ± 3^c^
*P* values	0.0003	<0.0001	<0.0001	<0.0001	0.0018	0.0004

^a–c^Treatment means with unequal superscripts are different at *P* < 0.05.

^1^Calculated from methane production rate (*μ*mol/mg cell DM/min) in percent of the value of the control group after 3 and 24 h, respectively.

^2^Percentage of live cells (green) of total cells (green, yellow, and red) as determined with the LIVE/DEAD BacLight Kit.

**Table 2:** Methane inhibition rate, K^+^ efflux, and cell viability in cell suspensions treated with different saturated fatty acids at 10 *μ*g/mL in Experiment 3 (*n* = 3; means ± standard error).

Time	3 h			24 h		
Treatment	K^+^(mg/L)	CH_4_ inhibition rate (%)^1^	Cell viability (%)^2^	K^+^ (mg/L)	CH_4_ inhibition rate (%)^1^	Cell viability (%)^2^
Blank	5.1 ± 0.1^d^	−6.8 ± 3.5^b^	79 ± 7^a^	14.2 ± 0.3^b^	−2.5 ± 11.1^b^	50 ± 8^a^
Control	5.4 ± 0.4^d^	—^b^	81 ± 2^a^	12.9 ± 0.3^b^	—^b^	40 ± 6^a^
C_12_	13.8 ± 0.2^a^	99.9 ± 0.0^a^	43 ± 2^b^	15.7 ± 0.2^a^	100 ± 0.1^a^	1 ± 0^b^
C_14_	13.9 ± 0.2^a^	99.8 ± 0.2^a^	36 ± 5^b^	15.7 ± 0.3^a^	100 ± 0.0^a^	3 ± 1^b^
C_16_	8.7 ± 0.2^b^	85.4 ± 1.8^a^	68 ± 6^a^	13.5 ± 0.1^b^	100 ± 0.1^a^	12 ± 5^b^
C_18_	7.0 ± 0.1^c^	7.9 ± 17.6^b^	78 ± 5^a^	13.9 ± 0.2^b^	44 ± 16.2^ab^	38 ± 5^a^
*P* values	<0.0001	<0.0001	<0.0001	<0.0001	0.0015	<0.0001

^a–d^Treatment means with unequal superscripts are different at *P* < 0.05.

^1^Calculated from methane production rate (*μ*mol/mg cell DM/min) in percent of the value of the control group after 3 and 24 h, respectively.

^2^Percentage of live cells (green) of total cells (green, yellow, and red) as determined with the LIVE/DEAD BacLight Kit.
