# Supplementary material for: The Effect of Saturated Fatty Acids on Methanogenesis and Cell Viability of Methanobrevibacter ruminantium
Source: Archaea. 2013 Apr 28;2013:106916. doi: 10.1155/2013/106916 (PMC3655487; doi:10.1155/2013/106916)
Supplement: Supplementary file 1 — Methane production rate (µmol/mg cell DM/min) in cell suspensions of M. ruminantium in K+-containing buffer (n = 3) in response to supplementation of different concentrations of lauric acid (A), myristic acid (B), palmitic acid (C) and stearic acid (D) at 37°C and of stearic acid at 50°C (E) in the second incubation series. Means within time point with unequal letters (a, b) are different at P < 0.05. Bars represent standard errors. [file 106916.f1.doc]

Supplementary Figure 1: Methane production rate (µmol/mg cell DM/min) in cell suspensions of *M. ruminantium* in K^+^-containing buffer (n = 3) in response to supplementation of different concentrations of lauric acid (A), myristic acid (B), palmitic acid (C) and stearic acid (D) at 37°C and of stearic acid at 50°C (E) in the second incubation series. Means within time point with unequal letters (a, b) are different at *P* < 0.05. Bars represent standard errors
